# Serial Cardiac Troponin I Kinetics Do Not Improve Detection of Acute Cellular Rejection Beyond Concurrent Troponin Levels After Heart Transplantation

**DOI:** 10.3390/jcm15166444

**Published:** 2026-08-20

**Authors:** Michał Ochman, Barbara Bilnik, Magdalena Cielecka, Michał Zakliczyński

**Affiliations:** 1 Student Scientific Club of Transplantology and Advanced Therapies of Heart Failure, Institute of Heart Diseases, Faculty of Medicine, Wroclaw Medical University, 50-556 Wroclaw, Poland; michal.ochman@student.umw.edu.pl (M.O.); barbara.bilnik@student.umw.edu.pl (B.B.); 2 Institute of Heart Diseases, Jan Mikulicz-Radecki University Hospital, 50-556 Wroclaw, Poland; 3 Clinical Department of Heart Transplantation and Mechanical Circulatory Support, Department of Cardiac Surgery and Heart Transplantation, Institute of Heart Diseases, Faculty of Medicine, Wroclaw Medical University, 50-556 Wroclaw, Poland

**Keywords:** heart transplantation, acute cellular rejection, cardiac troponin I, endomyocardial biopsy, diagnostic accuracy, biomarker kinetics

## Abstract

**Background/Objectives**: High-sensitivity cardiac troponin I (hs-cTnI) has shown inconsistent diagnostic performance for acute cellular rejection (ACR) after heart transplantation. We evaluated whether time-normalized hs-cTnI kinetics provide incremental diagnostic value beyond the concurrent concentration for detecting ACR grade ≥ 2R and whether this association varies with time after transplantation. **Methods**: This retrospective single-center study included 1237 biopsy episodes from 139 heart transplant recipients. Two generalized linear mixed-effects logistic regression models with patient-specific random intercepts were compared. M1 included concurrent log_2_-transformed hs-cTnI, whereas M2 additionally included the time-normalized change in log_2_-transformed hs-cTnI per 7 days. Discrimination was assessed using leave-one-patient-out cross-validation with patient-level cluster bootstrap confidence intervals. Exploratory analyses examined early (≤90 days), late (>90 days), and continuous post-transplant time. **Results**: In the overall cohort, M1 showed modest discrimination (AUC 0.641, 95% CI 0.592–0.689), whereas M2 provided no improvement (AUC 0.635; ΔAUC −0.007). Within 90 days, neither model was informative (M1 AUC 0.503; M2 AUC 0.494). Beyond 90 days, M1 showed moderate discrimination (AUC 0.758, 95% CI 0.664–0.838), but M2 again provided no improvement (AUC 0.746; ΔAUC −0.012). Continuous-time modeling showed that the association between concurrent hs-cTnI and ACR strengthened with increasing time after transplantation, whereas the kinetic term remained non-informative. **Conclusions**: Time-normalized hs-cTnI kinetics did not provide incremental diagnostic value beyond concurrent hs-cTnI. Concurrent hs-cTnI may warrant further evaluation as an adjunctive late-period marker, but the 90-day threshold remains exploratory and requires external validation. Neither hs-cTnI concentration nor its kinetics should replace endomyocardial biopsy.

## 1. Introduction

Acute cellular rejection (ACR) remains an important complication after orthotopic heart transplantation (OHT), particularly during the first post-transplant year. Contemporary UNOS registry data indicate that approximately 22.7% of heart transplant recipients experience rejection requiring treatment during the first year after transplantation [1]. Although this endpoint encompasses treated rejection rather than exclusively biopsy-confirmed ACR grade ≥ 2R, it highlights the continuing clinical burden of post-transplant rejection despite advances in immunosuppressive therapy. Endomyocardial biopsy (EMB) remains the reference standard for surveillance and diagnosis of ACR [2,3]. However, repeated EMB is invasive, resource-intensive, associated with procedural risk, and subject to sampling and interpretative variability. Moreover, most surveillance biopsies do not demonstrate clinically significant rejection [2,3]. These limitations provide a strong rationale for developing inexpensive and widely available non-invasive markers that could complement EMB and improve the selection of patients requiring invasive testing.

Cardiac troponin I (cTnI) is a cardiac-specific marker of myocardial injury and has therefore been investigated as a potential biomarker of cardiac allograft rejection. However, cardiac specificity does not imply specificity for rejection, as troponin may also increase in response to perioperative myocardial injury and other non-rejection causes of myocardial damage. Previous studies evaluating cardiac troponin, including high-sensitivity cardiac troponin I (hs-cTnI), for ACR have yielded inconsistent results [4,5,6,7]. Some studies have suggested potentially useful rule-out properties, particularly because of high negative predictive values, whereas larger observational studies and meta-analyses have demonstrated limited overall diagnostic discrimination [4,5,6,7]. Current ISHLT guidance does not support troponin as a stand-alone strategy for routine rejection surveillance [3], and the European Society for Organ Transplantation has similarly concluded that the available evidence is insufficient to support routine troponin-based diagnosis of ACR [2]. Thus, although hs-troponin is inexpensive and widely available, its role in rejection surveillance remains uncertain, providing a rationale for determining whether additional information derived from serial measurements could improve its diagnostic performance.

The early postoperative period is particularly challenging because cardiac troponin release may remain elevated independently of rejection [8]. Perioperative myocardial injury and the effects of transplantation itself may therefore reduce the specificity of troponin for ACR during this period [8]. Previous studies have consequently restricted troponin assessment to later post-transplant periods or otherwise accounted for the immediate postoperative phase when evaluating its diagnostic value [9,10,11]. This provides a biological and literature-based rationale for examining whether the diagnostic information carried by troponin changes with time from transplantation.

Most previous studies have focused primarily on a single troponin concentration [4,5,6,7,9,10,11]. Whether serial changes in hs-cTnI provide incremental information beyond the concurrent concentration remains uncertain. We therefore evaluated whether a time-normalized change in log_2_-transformed hs-cTnI improves discrimination for biopsy-confirmed ACR grade ≥ 2R beyond the concurrent hs-cTnI concentration. The principal novelty of the present study was the direct comparison of these two approaches while accounting for repeated measurements within recipients and evaluating predictive performance using patient-level cross-validation.

## 2. Methods

### 2.1. Study Design and Population

We conducted a retrospective, single-center diagnostic cohort study of post-HTx patients, evaluating whether troponin dynamics add to absolute troponin levels in predicting biopsy-confirmed ACR graded ≥ 2R according to ISHLT criteria. Endomyocardial biopsies were originally graded according to the 1990 ISHLT classification [12] and were subsequently converted to the revised ISHLT grading system [13] for the purposes of the present analysis. According to the established correspondence between the two classifications, former grades 1A, 1B, and 2 were classified as grade 1R, former grade 3A as grade 2R, and former grades 3B and 4 as grade 3R; grade 0 corresponded to grade 0R. Accordingly, the primary study endpoint of ACR grade ≥ 2R included biopsy episodes originally classified as grade 3A or higher. In the revised ISHLT classification, grade 2R represents moderate acute cellular rejection, characterized by two or more foci of inflammatory infiltrates with associated myocyte damage, whereas grade 3R represents severe acute cellular rejection. The study was designed as a diagnostic accuracy study comparing concurrent hs-cTnI testing (index test) with EMB (reference standard). The study was conducted at the Department of Heart Transplantation and Cardiac Surgery of the University Clinical Hospital in Wrocław. The pathologists assessing endomyocardial biopsies were blinded to the corresponding cTnI results. For the purposes of this study, concurrent cTnI was defined as a cTnI measurement obtained on the same calendar day as the corresponding endomyocardial biopsy. Laboratory personnel performing cTnI measurements were blinded to the biopsy results. Data from 141 recipients, comprising 1392 biopsy–troponin pairs, were initially screened. Biopsy episodes with ambiguous multiple cTnI measurements recorded for the same patient and date were excluded. For the comparative model analysis, only episodes with a concurrent cTnI measurement and a preceding confirmed cTnI measurement were retained. The final analytical dataset comprised 1237 biopsy episodes from 139 recipients.

All consecutive OHT recipients with eligible biopsy–troponin pairs during the study period were considered for inclusion. No patient-level clinical exclusion criteria were applied. In particular, recipients were not excluded because of renal dysfunction, infection, hemodynamic instability, graft dysfunction, treatment modifications, or other potential causes of myocardial injury. This all-comers approach was deliberately chosen to evaluate the diagnostic performance of cTnI under routine clinical conditions representative of its intended clinical use.

Exclusions were applied only at the biopsy-episode level. Episodes were excluded when multiple cTnI values recorded for the same patient and calendar date prevented unambiguous assignment of a single cTnI result to the corresponding biopsy. For the comparative analysis of concurrent cTnI and cTnI kinetics, episodes without an available preceding confirmed cTnI measurement were also excluded because the kinetic term could not be calculated. The final comparative analytical cohort comprised 1237 biopsy episodes from 139 recipients.

### 2.2. Data Collection

Clinical, laboratory, and histopathological data were collected between February 2021 and March 2025 based on the analysis of electronic medical records and the results of laboratory and histopathological tests. Endomyocardial biopsies were assessed according to the ISHLT classification.

The analytical dataset included recipient age at OHT, sex, transplantation date, biopsy date and histopathological rejection grade, and the hs-cTnI concentration obtained on the same calendar day as the corresponding EMB. For the analysis of hs-cTnI kinetics, the preceding eligible hs-cTnI concentration and the exact interval between the two hs-cTnI measurements were additionally recorded.

High-sensitivity cardiac troponin I (hs-cTnI) was measured using the Alinity i STAT High Sensitive Troponin-I assay (Abbott Laboratories, Abbott Park, IL, USA) on the Alinity i immunoassay platform. The assay is a two-step chemiluminescent microparticle immunoassay (CMIA). According to the manufacturer, the limit of blank, limit of detection, and limit of quantitation are 1.0, 1.6, and 5.1 pg/mL, respectively, and the analytical measurement range is 10–50,000 pg/mL. The same analyzer and assay were used throughout the entire study period, with no change in the analytical platform.

Endomyocardial biopsies and paired hs-cTnI measurements followed the routine institutional post-transplant surveillance schedule rather than fixed study-defined intervals. Surveillance intensity was highest immediately after transplantation and decreased progressively over time. In the analytical cohort, the median interval between consecutive biopsy assessments was approximately 7 days (IQR 6–7) during the first 30 days after OHT, 16 days (IQR 14–19) during days 31–60, 27 days (IQR 20–31) during days 61–120, 59 days (IQR 49–63) during days 121–365, and 182 days (IQR 64–183) thereafter. This broadly corresponded to weekly surveillance during the first month, approximately biweekly surveillance during the second month, monthly surveillance during the subsequent early period, and progressively less frequent surveillance thereafter. Additional clinically indicated or follow-up biopsies and routine scheduling variation resulted in non-uniform sampling intervals.

Post-transplant maintenance immunosuppression was managed according to the institutional standard-of-care protocol consistent with contemporary international recommendations. Maintenance therapy was based on a calcineurin inhibitor, generally tacrolimus, combined with an antiproliferative agent, generally mycophenolate, with corticosteroids used during the early post-transplant period and tapered as clinically appropriate. Individual modifications were made according to clinical status, rejection history, treatment tolerance, and other patient-specific considerations.

Biopsy-confirmed significant ACR, both asymptomatic and symptomatic, was treated with intravenous corticosteroid therapy and optimization of maintenance immunosuppression; intensified therapy was used in clinically significant, recurrent, or treatment-resistant rejection as appropriate.

Detailed episode-level data on renal function, active infection, hemodynamic parameters, ventricular function, electrocardiographic findings, and other concurrent causes of myocardial injury were not systematically available for the complete retrospective cohort and were therefore not included as covariates in the primary models. No imputation of unavailable clinical covariates was performed.

### 2.3. Statistical Analysis

All analyses were performed in R version 4.5.1 (R Foundation for Statistical Computing, Vienna, Austria) using the lme4 and pROC packages. The biopsy episode was considered the unit of analysis. Because multiple biopsy episodes were available for individual recipients, generalized linear mixed-effects models with a binomial distribution, logit link, and a patient-specific random intercept were used.

Only biopsy episodes with a concurrent hs-cTnI measurement and an available preceding hs-cTnI measurement were included in the head-to-head comparison of the two models. Consequently, the first biopsy episode of each patient was excluded from the primary analysis. Both models were fitted and evaluated using the same complete-case analytical dataset.

Concurrent cTnI concentrations were transformed using the base-2 logarithm. Troponin kinetics were represented by the time-normalized change in log-transformed cTnI:

$$K_{i}=7\times \frac{{log}_{2}\left(cTnI_{i}\right)-{log}_{2}\left(cTnI_{i-1}\right)}{\Delta t_{i}},$$

where $cTnI_{i}$ and $cTnI_{i-1}$ denote the current and preceding cTnI concentrations, respectively, and $\Delta t_{i}$ denotes the exact interval between the two measurements in days. The variable was expressed per 7 days to facilitate interpretation. Positive values represented an increase in cTnI, negative values represented a decrease, and a value of zero represented no change.

Two nested mixed-effects logistic regression models were evaluated. The baseline model (M1) included the concurrent log_2_-transformed cTnI concentration as the only fixed-effect predictor. The extended model (M2) additionally included the time-normalized log_2_ cTnI change:

$$M1:ACR\geq 2R∼{log}_{2}\left(cTnI\right)+\left(1|patient\right),M2:ACR\geq 2R∼{log}_{2}\left(cTnI\right)+K_{i}+\left(1|patient\right).$$

Predictive discrimination was assessed using leave-one-patient-out cross-validation. At each iteration, all biopsy episodes belonging to one recipient were excluded, the model was fitted using the remaining recipients, and population-level predicted probabilities were generated for the excluded recipient. This procedure ensured that no observations from the same recipient were present simultaneously in the training and validation datasets.

Discrimination was quantified using the empirical area under the receiver operating characteristic curve. The primary analysis included all eligible biopsy episodes. Discrimination was also assessed exploratorily in biopsies performed within 90 days after transplantation and in biopsies performed more than 90 days after transplantation, using the same cross-validated predictions.

Ninety-five percent confidence intervals for the AUCs and for the paired difference in AUC between M2 and M1 were obtained using 2000 patient-level cluster bootstrap resamples. Patients were sampled with replacement, and all biopsy episodes belonging to each sampled patient were retained. The difference in AUC was calculated as AUC _M2_−AUC _M1_. Full-dataset models were fitted separately to report fixed-effect estimates as log-odds coefficients, odds ratios with 95% Wald confidence intervals, and patient-level random-intercept variance. Fixed-effect *p* values were derived from Wald z tests. The nested full-data models were compared using a likelihood-ratio test. All reported measures of predictive discrimination were based on leave-one-patient-out cross-validated predictions.

In addition to discrimination, predictive performance was evaluated using the Brier score and calibration. Calibration was summarized using the calibration intercept based on leave-one-patient-out cross-validated predicted probabilities. Diagnostic classification performance was additionally summarized using sensitivity, specificity, positive predictive value, and negative predictive value across clinically interpretable probability thresholds. The corresponding numbers of true-positive, false-positive, true-negative, and false-negative classifications were reported. Clinical utility was explored using decision-curve analysis, comparing the net benefit of M1 and M2 with strategies of biopsy in all patients and biopsy in no patients over a range of threshold probabilities. Where confidence intervals were reported, patient-level cluster bootstrap resampling was used to preserve within-recipient correlation.

Exploratory predicted-risk thresholds of 2%, 5%, 10%, 15%, and 20% were evaluated. These thresholds were selected for descriptive clinical interpretation and were not optimized on the observed outcomes. Decision-curve analysis was performed over threshold probabilities ranging from 1% to 25%.

For the prespecified exploratory stratified analysis, the early post-transplant period was defined as ≤90 days after OHT and the late period as >90 days after OHT. The 90-day landmark was selected on biological and literature-based grounds rather than being derived from the observed study AUCs: postoperative troponin release may persist for several weeks after OHT [8], and previous studies evaluating troponin for cardiac allograft rejection have used or focused on a three-month post-transplant landmark [9,10,11]. Accordingly, the stratified analyses were considered exploratory and hypothesis-generating.

To avoid relying exclusively on a dichotomous 90-day cutoff, an additional exploratory analysis modeled time since OHT continuously. Post-transplant time was log_2_-transformed and centered at 90 days, and an interaction between concurrent log_2_-transformed hs-cTnI and continuous post-transplant time was evaluated in a mixed-effects logistic regression model with a patient-specific random intercept. The interaction was used to assess whether the association between hs-cTnI and ACR varied across post-transplant time.

An extended continuous-time model additionally included the time-normalized hs-cTnI kinetic term, and its incremental contribution was assessed by comparison with the interaction model using a likelihood-ratio test.

## 3. Results

### 3.1. Baseline Comparisons

The original dataset comprised 1392 biopsy–troponin pairs from 141 heart transplant recipients. After conservative exclusion of ambiguous measurements and biopsy episodes without a preceding confirmed cTnI result, 1237 biopsy episodes from 139 recipients were included in the comparative model analysis. ACR grade ≥ 2R was present in 128 biopsy episodes (10.3%).

The early-period analysis included 625 biopsies from 138 recipients, of which 73 (11.7%) demonstrated ACR ≥ 2R. The late-period analysis included 612 biopsies from 126 recipients, of which 55 (9.0%) demonstrated ACR ≥ 2R (Table 1).

### 3.2. Overall Discrimination

**Figure 1:**
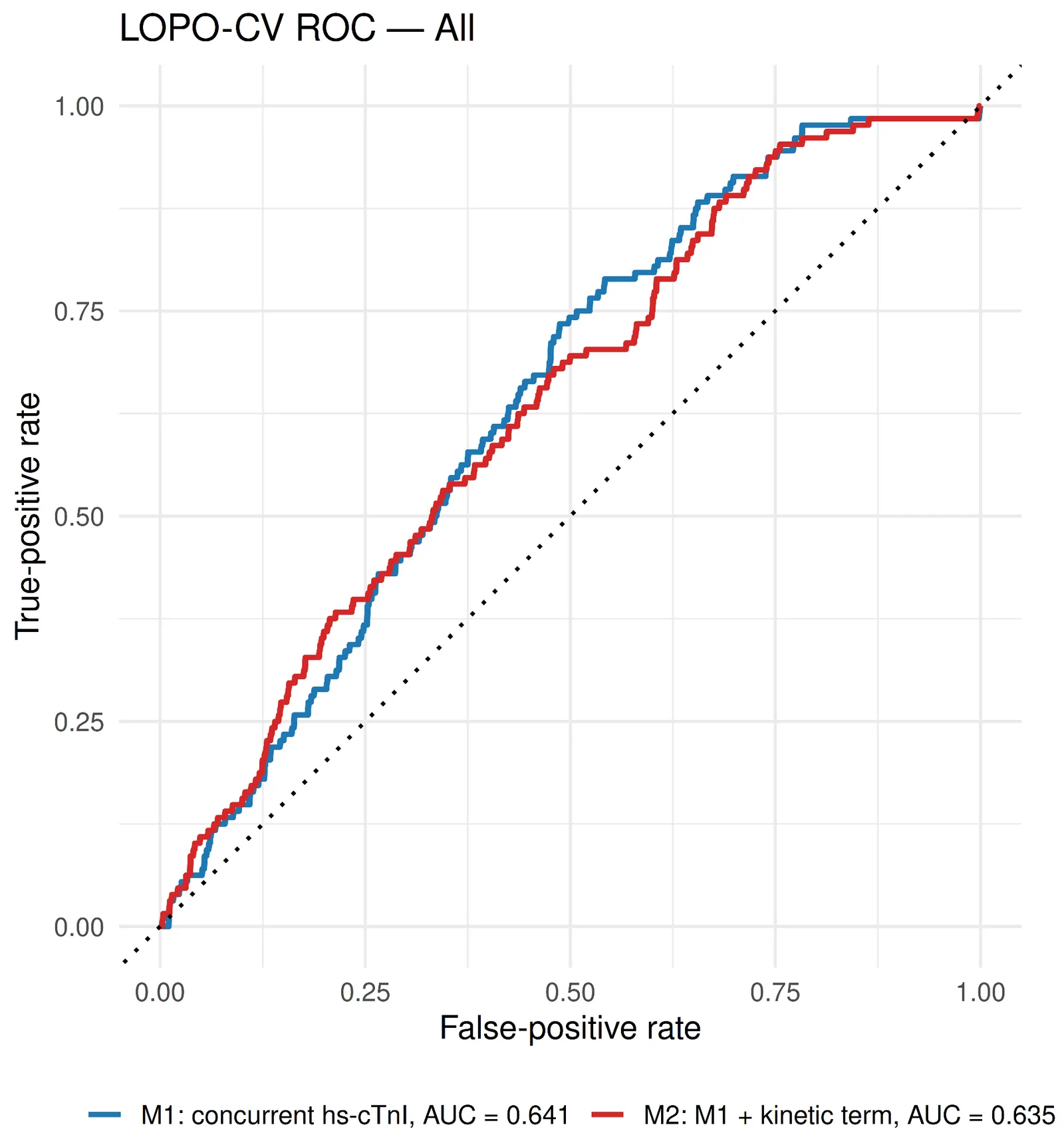
Leave-one-patient-out cross-validated receiver operating characteristic (ROC) curves for M1 and M2 in the overall analytical cohort. The dotted diagonal line represents the no-discrimination reference line (AUC = 0.5).

In the overall cohort, concurrent log_2_-transformed cTnI demonstrated modest discrimination for ACR ≥ 2R (Figure 1). The leave-one-patient-out cross-validated AUC for M1 was 0.641 (95% CI 0.592–0.689).

Addition of the time-normalized cTnI kinetic term did not improve discrimination. The corresponding AUC for M2 was 0.635 (95% CI 0.584–0.685), with a paired ΔAUC of −0.007 (95% CI −0.021 to 0.007).

### 3.3. Early Post-Transplant Period

**Figure 2:**
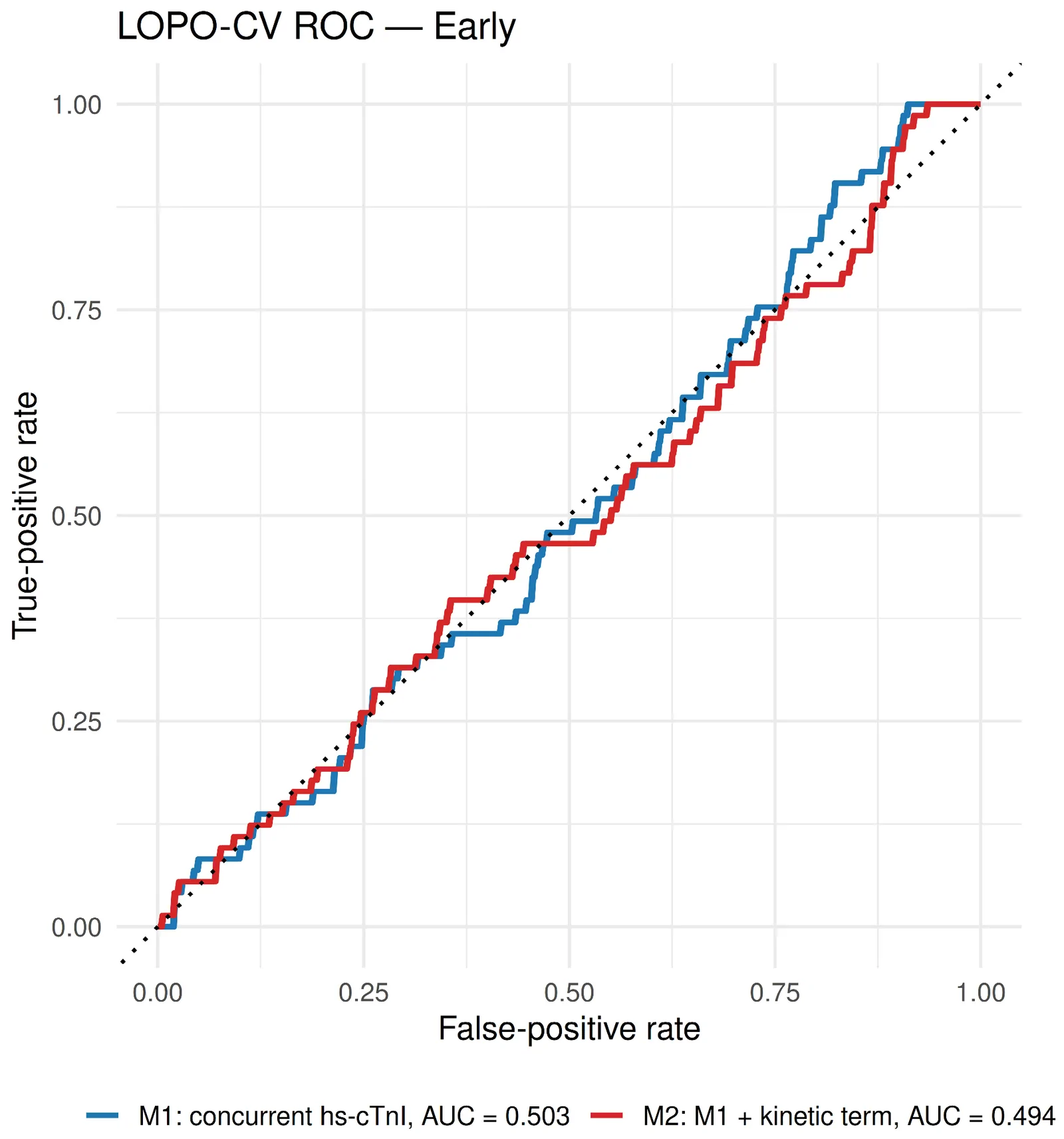
Leave-one-patient-out cross-validated ROC curves for M1 and M2 during the early post-transplant period (≤90 days after OHT). The dotted diagonal line represents the no-discrimination reference line (AUC = 0.5).

During the first 90 days after transplantation, neither model demonstrated meaningful discrimination (Figure 2). The AUC was 0.503 (95% CI 0.439–0.573) for M1 and 0.494 (95% CI 0.425–0.568) for M2. The paired ΔAUC was −0.009 (95% CI −0.032 to 0.013), indicating no incremental value from the kinetic term. 

### 3.4. Late Post-Transplant Period

In the exploratory analysis beyond 90 days after transplantation, concurrent hs-cTnI demonstrated moderate discrimination (M1 AUC 0.758, 95% CI 0.664–0.838).

**Figure 3:**
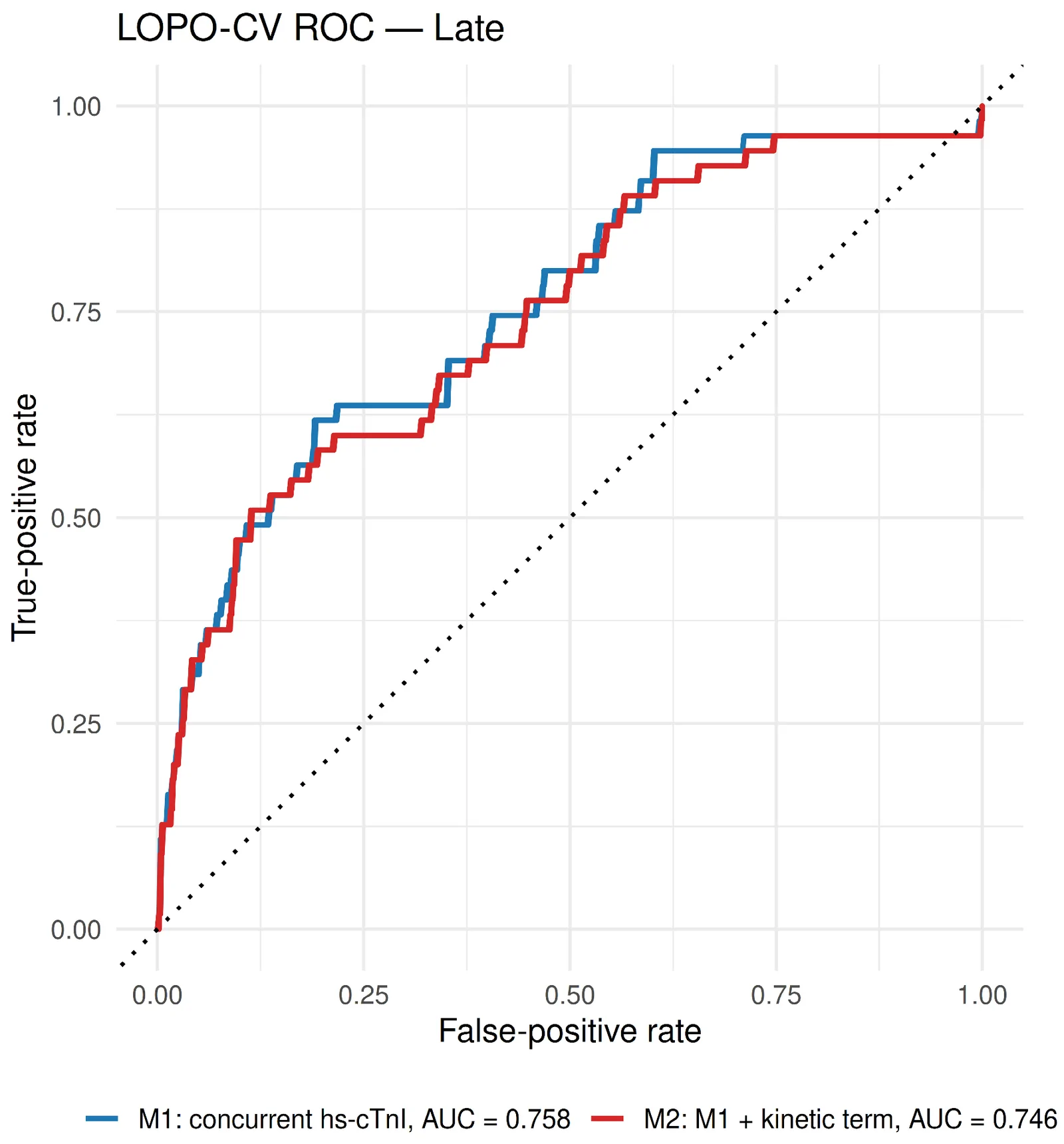
Leave-one-patient-out cross-validated ROC curves for M1 and M2 during the late post-transplant period (>90 days after OHT). The dotted diagonal line represents the no-discrimination reference line (AUC = 0.5).

Addition of the kinetic term did not improve performance (Figure 3). The AUC for M2 was 0.746 (95% CI 0.654–0.828), with a paired ΔAUC of −0.012 (95% CI −0.037 to 0.008). 

Given the exploratory nature of this subgroup analysis, the relatively wide confidence interval, and the absence of a validated clinical threshold, the late-period estimate should be regarded as hypothesis-generating.

### 3.5. Mixed-Effects Models and Internal Validation

Both models were fitted using patient-specific random intercepts, and neither model demonstrated a singular fit. Predictive performance was evaluated exclusively using leave-one-patient-out cross-validated probabilities. All 2000 patient-level cluster bootstrap resamples yielded valid AUC estimates (Table 2).

In the full-data mixed-effects models, concurrent log_2_-transformed cTnI was positively associated with ACR grade ≥ 2R. In M1, each two-fold increase in concurrent cTnI was associated with a 21.4% increase in the odds of ACR ≥ 2R (OR 1.214, 95% CI 1.127–1.309; *p* < 0.001). After addition of the kinetic term in M2, concurrent cTnI remained independently associated with ACR ≥ 2R (OR 1.234, 95% CI 1.139–1.336; *p* < 0.001). In contrast, the time-normalized kinetic term was not independently associated with ACR ≥ 2R (OR 1.099, 95% CI 0.935–1.293; *p* = 0.253). Addition of the kinetic term did not significantly improve full-model fit compared with M1 (likelihood-ratio χ^2^(1) = 1.326; *p* = 0.249). Patient-level random-intercept variance was 0.431 in M1 and 0.446 in M2, corresponding to standard deviations of 0.657 and 0.668, respectively (Table 3).

### 3.6. Continuous-Time Analysis

When post-transplant time was modeled continuously, the association between concurrent log_2_-transformed hs-cTnI and ACR grade ≥ 2R varied significantly with time since OHT. The hs-cTnI × post-transplant time interaction was positive (β = 0.141, SE = 0.030; Wald *p* < 0.001), and addition of the interaction significantly improved model fit (likelihood ratio χ^2^(1) = 28.115; *p* < 0.001). The estimated odds ratio for ACR grade ≥ 2R associated with a two-fold increase in hs-cTnI was 1.089 (95% CI 0.961–1.235; *p* = 0.182) at 30 days, 1.362 (95% CI 1.213–1.530; *p* < 0.001) at 90 days, 1.569 (95% CI 1.358–1.813; *p* < 0.001) at 180 days, and 1.812 (95% CI 1.502–2.187; *p* < 0.001) at 365 days after OHT. Addition of the kinetic term to the continuous-time interaction model did not improve fit (likelihood ratio χ^2^(1) = 0.134; *p* = 0.714), and the kinetic term remained non-significant (OR 0.963, 95% CI 0.785–1.180; *p* = 0.714). None of the continuous-time mixed-effects models showed a singular fit (Table 4). These findings support a time-varying association between concurrent hs-cTnI and rejection, while providing no evidence that hs-cTnI kinetics add incremental information after accounting for post-transplant time. Because this analysis was exploratory, the findings should be considered hypothesis-generating.

### 3.7. Calibration and Clinical Utility

Calibration and overall prediction error were similar for the two models (Table 5). In the overall cohort, the Brier score was 0.0917 (95% CI 0.0758–0.1082) for M1 and 0.0917 (95% CI 0.0759–0.1083) for M2. Calibration intercepts were 0.165 (95% CI −0.059 to 0.373) and 0.170 (95% CI −0.055 to 0.379), while calibration slopes were 0.869 (95% CI 0.559–1.213) and 0.813 (95% CI 0.498–1.149), respectively. Calibration was strongly period-dependent. During the early period, calibration slopes were close to zero (M1: 0.093, 95% CI −0.399 to 0.591; M2: 0.023, 95% CI −0.472 to 0.496), consistent with limited predictive information. In the late period, calibration slopes were substantially above one (M1: 2.454, 95% CI 1.689–3.415; M2: 2.122, 95% CI 1.449–2.956), indicating period-specific miscalibration despite better discrimination.

Calibration plots are presented in Supplementary Figures S1–S3.

At an exploratory predicted-risk threshold of 5%, M1 achieved 97.7% sensitivity (95% CI 93.6–100.0%), 19.7% specificity (95% CI 15.0–24.3%), 12.3% positive predictive value (95% CI 10.1–14.5%), and 98.6% negative predictive value (95% CI 96.4–100.0%) in the overall cohort (Table 6). This threshold would classify 17.9% of biopsy episodes as test-negative (95% CI 13.7–22.2%), while missing 3 of 128 ACR grade ≥ 2R episodes (2.3%; 95% CI 0–6.4%). M2 showed similar but slightly less favorable rule-out performance: sensitivity 96.1% (95% CI 91.9–99.2%), specificity 21.1% (95% CI 16.5–25.8%), PPV 12.3% (95% CI 10.1–14.6%), and NPV 97.9% (95% CI 95.6–99.6%), with 19.3% of biopsies potentially avoided (95% CI 15.0–23.6%) and 5 of 128 ACR episodes missed (3.9%; 95% CI 0.8–8.1%). In the early period, both models achieved 100% sensitivity and 100% NPV at the 5% threshold, but only 2.4% (95% CI 1.0–4.2%) and 3.2% (95% CI 1.5–5.3%) of biopsies, respectively, would have been avoided. In the late period, M1 achieved 94.5% sensitivity (95% CI 85.7–100.0%) and 98.6% NPV (95% CI 96.2–100.0%) while potentially avoiding 33.8% of biopsies (95% CI 26.0–41.4%); M2 achieved 90.9% sensitivity (95% CI 81.3–98.3%) and 97.7% NPV (95% CI 95.2–99.6%) while potentially avoiding 35.8% of biopsies (95% CI 28.4–43.4%). These thresholds were exploratory and were not externally validated.

Diagnostic performance across all exploratory predicted-risk thresholds of 2%, 5%, 10%, 15%, and 20% is presented in Supplementary Table S1.

Decision-curve analysis showed no consistent clinical-utility advantage of M2 over M1. In the early period, net-benefit differences between the models and the biopsy-all strategy were minimal. In the late period, both models showed greater net benefit than biopsy-all or biopsy-none across a broader intermediate threshold range, but the kinetic model did not consistently outperform the concurrent hs-cTnI model. These findings support the primary conclusion that adding hs-cTnI kinetics does not provide meaningful incremental value.

## 4. Discussion

The present study yielded four principal findings. First, concurrent cTnI demonstrated modest discrimination for biopsy-confirmed ACR ≥ 2R in the overall cohort. Second, its performance differed markedly according to time from transplantation: cTnI was non-informative during the first 90 days but showed moderate discrimination thereafter. Third, addition of the time-normalized change in cTnI did not improve discrimination in the overall, early, or late analyses. The point estimates for M2 were slightly lower than those for M1 in all three analyses, and all confidence intervals for ΔAUC included zero. Consistently, concurrent cTnI was independently associated with ACR ≥ 2R in the full-data mixed-effects models, whereas the kinetic term was not, and its addition did not significantly improve model fit. Fourth, continuous-time modeling demonstrated that the association between concurrent hs-cTnI and ACR strengthened with time after transplantation, whereas calibration and decision-curve analyses suggested potentially useful late-period rule-out characteristics without demonstrating incremental benefit from the kinetic model.

Previous studies evaluating cardiac troponin for the detection of ACR after heart transplantation have produced markedly heterogeneous results. One of the more favorable reports was published by Patel et al., who evaluated high-sensitivity cardiac troponin I and reported a c-statistic of approximately 0.82 (95% CI 0.76–0.88) for detection of acute rejection [7]. At a cutoff of 15 ng/L, the negative predictive value approached 99%, suggesting that hs-cTnI might potentially serve as a low-cost rule-out marker in selected patients. Similarly, the systematic review by Fitzsimons et al. reported that studies using high-sensitivity cTnI assays achieved sensitivities ranging from approximately 82% to 100% and negative predictive values of 97% to 100% [6]. These early observations provided an important rationale for further evaluation of troponin-based surveillance strategies.

More recent evidence, however, has been considerably less optimistic. In the systematic review and meta-analysis by Liu et al., the pooled overall diagnostic performance of cardiac troponin for ACR was poor, with a Bayesian AUC of 0.584 [5]. Even after exclusion of measurements obtained during the first postoperative month, when perioperative myocardial injury is likely to have the greatest influence on circulating troponin, diagnostic performance remained limited, with a Bayesian AUC of approximately 0.607. In a large single-center study including 170 heart transplant recipients and 883 paired hs-cTnI–biopsy observations, Fitzsimons et al. identified 51 episodes of significant ACR and reported an overall AUC of only 0.509 (95% CI 0.428–0.591) [4]. Importantly, performance remained essentially equivalent to chance in their early post-transplant analysis, with an AUC of approximately 0.51. Thus, although earlier studies suggested that low cTnI concentrations might have useful rule-out properties, subsequent larger analyses have not consistently reproduced sufficient discrimination for cTnI to function as a stand-alone rejection marker.

Our findings fall between these previously reported extremes and further demonstrate the importance of post-transplant timing. In the present cohort of 139 recipients and 1237 biopsy episodes, concurrent hs-cTnI showed modest overall discrimination, with a patient-level cross-validated AUC of 0.641 (95% CI 0.592–0.689). This value is higher than the AUC of 0.509 reported by Fitzsimons et al. [4] and the pooled Bayesian AUC of 0.584 reported by Liu et al. [5], but remains substantially below the c-statistic of approximately 0.82 described by Patel et al. [7]. The apparent discrepancy was largely related to time since transplantation. During the first 90 days, hs-cTnI was essentially non-informative in our cohort, with an AUC of 0.503 (95% CI 0.439–0.573), closely resembling the near-chance early performance reported by Fitzsimons et al. In contrast, beyond 90 days the AUC increased to 0.758 (95% CI 0.664–0.838), approaching, although not reaching, the discrimination reported in the earlier study by Patel et al. This late-period result should nevertheless be interpreted cautiously because the analysis was exploratory, the confidence interval was relatively wide, and no clinical cutoff was externally validated.

The additional continuous-time analysis strengthens the interpretation that the diagnostic association of concurrent hs-cTnI changes progressively with time after transplantation rather than abruptly at an arbitrary 90-day threshold. The association between hs-cTnI and ACR significantly increased with post-transplant time (hs-cTnI × time interaction, likelihood-ratio *p* < 0.001). The estimated odds ratio for ACR grade ≥ 2R associated with each two-fold increase in hs-cTnI was 1.09 (95% CI 0.96–1.24) at 30 days, 1.36 (95% CI 1.21–1.53) at 90 days, 1.57 (95% CI 1.36–1.81) at 180 days, and 1.81 (95% CI 1.50–2.19) at 365 days. These findings provide a plausible explanation for some of the heterogeneity between previous studies, because cohorts differing in the timing of troponin sampling may yield substantially different estimates of diagnostic performance. They also argue against interpreting a single pooled AUC as a fixed property of hs-cTnI across the entire post-transplant course. Most recently, Scussel et al. evaluated 187 biopsies obtained between 3 months and 2 years after HT and reported an AUC of 0.705 for hs-cTnI, with 88% sensitivity, 49% specificity, and a negative predictive value of 92% at a cutoff of 19 ng/L [11]. These results are broadly consistent with the moderate late-period discrimination observed in our cohort (AUC 0.758), while further supporting the importance of post-transplant timing.

Most importantly, our study extends the existing literature by directly testing whether the preceding hs-cTnI trajectory contains diagnostic information beyond the concurrent concentration. It did not. In the overall analysis, adding the time-normalized kinetic term decreased rather than improved the cross-validated AUC from 0.641 to 0.635 (ΔAUC −0.007, 95% CI −0.021 to 0.007). The same pattern was observed during both the early period (0.503 vs. 0.494; ΔAUC −0.009) and the late period (0.758 vs. 0.746; ΔAUC −0.012). In the full-data mixed-effects model, the kinetic term was also not independently associated with ACR (OR 1.099, 95% CI 0.935–1.293; *p* = 0.253), and its addition did not improve model fit (likelihood-ratio *p* = 0.249). Furthermore, after explicitly accounting for the continuous interaction between concurrent hs-cTnI and post-transplant time, addition of the kinetic term again failed to improve model fit (likelihood-ratio *p* = 0.714). Taken together, these findings suggest that variation between previously reported troponin studies is more likely to reflect differences in post-transplant timing, assay characteristics, patient selection, rejection prevalence, and study design than a clinically useful contribution from short-term troponin kinetics themselves.

Troponin trajectories may also be affected by renal function, infection, hemodynamic instability, non-rejection myocardial injury, and changes in treatment. Consequently, a rise or fall in cTnI is not specific to rejection. These sources of variability may outweigh any additional signal provided by the kinetic term. Our findings therefore do not support the routine use of serial hs-cTnI kinetics as a means of improving rejection detection beyond the concurrent concentration.

The time-dependent performance of cTnI is biologically plausible. During the early post-transplant period, cTnI concentrations are influenced by perioperative myocardial injury [8], ischemia–reperfusion injury, surgical manipulation, hemodynamic instability, and other non-rejection-related processes. These competing sources of cardiomyocyte injury may explain why concurrent hs-cTnI was essentially non-informative during the first 90 days.

Beyond 90 days, the effect of perioperative injury is expected to diminish. In this setting, an elevated hs-cTnI concentration may more specifically reflect new myocardial injury, including injury associated with rejection. This may partly explain the late-period AUC of 0.758 observed in the present cohort. At the exploratory 5% predicted-risk threshold, M1 achieved a sensitivity of 94.5% and an NPV of 98.6%, while potentially classifying 33.8% of late-period biopsy episodes as test-negative. However, these findings should not be interpreted as evidence that hs-cTnI can replace biopsy. The threshold was exploratory and not externally validated, late-period calibration remained imperfect, and decision-curve analysis did not demonstrate a consistent clinical-utility advantage from adding hs-cTnI kinetics. Thus, concurrent hs-cTnI may warrant further prospective evaluation as an adjunctive late-period rule-out marker, but it cannot currently be used to defer EMB.

The limited performance of troponin as a stand-alone marker should be considered in the broader context of evolving non-invasive rejection surveillance. Contemporary approaches include donor-derived cell-free DNA, gene-expression profiling, natriuretic peptides, microRNA and extracellular-vesicle signatures, as well as multimarker and multi-omics strategies [14]. Recent perspectives therefore favor risk-adapted and multimodal surveillance strategies rather than simple replacement of EMB by a single biomarker [14,15]. Donor-derived cell-free DNA is particularly attractive as a rule-out tool because of its high negative predictive value in selected populations [16], whereas emerging approaches such as extracellular-vesicle profiling remain promising but require larger external validation cohorts [17].

Echocardiography represents another important non-invasive component of follow-up. Conventional ventricular function may remain preserved during clinically relevant rejection, whereas advanced echocardiographic techniques, particularly tissue Doppler and myocardial deformation imaging, may identify subtle graft dysfunction that is not apparent from conventional measures of ventricular function [18]. Combining changes in global longitudinal strain with cardiac biomarkers may further improve non-invasive rule-out performance [19]. Taken together, these data support a multimodal strategy rather than replacement of EMB by any single conventional biomarker.

From a clinical perspective, our findings do not support using serial hs-cTnI kinetics to determine whether a surveillance or clinically indicated EMB should be omitted. During the first 90 days, neither the concurrent concentration nor the kinetic term provided meaningful discrimination. Beyond 90 days, concurrent hs-cTnI may be considered only as an adjunctive marker within a broader clinical assessment, and the exploratory late-period findings require confirmation. A low or decreasing hs-cTnI concentration should therefore not be used in isolation to defer a clinically indicated biopsy.

### Limitations

This study has several limitations. First, it was a retrospective, single-center analysis, which may limit external generalizability. Second, the study deliberately used an all-comers OHT population without patient-level clinical exclusions for conditions potentially affecting hs-cTnI. Detailed episode-level information on renal function, including creatinine and estimated glomerular filtration rate, active infections, hemodynamic parameters, ventricular function, electrocardiographic findings, and other concurrent causes of myocardial injury, was not systematically available and could therefore not be incorporated into adjusted models. Detailed longitudinal information on individual immunosuppressive drug doses, trough concentrations, and temporary treatment modifications was likewise not available for every biopsy episode. This design improves representativeness of routine clinical practice but introduces residual confounding and may reduce the specificity of hs-cTnI for rejection.

Third, the number of moderate-or-greater rejection episodes limited precision, particularly in stratified analyses. Fourth, biopsy and hs-cTnI sampling intervals were not standardized because measurements followed routine post-transplant surveillance, with progressively longer intervals after transplantation and additional clinically indicated assessments. Although the kinetic variable was normalized to the exact elapsed time, a rate calculated from two measurements may be unstable when measurements are separated by long intervals or when the underlying trajectory is nonlinear.

Fifth, all hs-cTnI measurements were obtained using a single high-sensitivity assay platform. This ensured analytical consistency within the cohort but may limit transferability of absolute concentrations or diagnostic thresholds to other assays. Finally, the ≤90-day and >90-day analyses were exploratory, and the 90-day landmark should not be interpreted as a validated clinical cutoff. Although the additional continuous-time analysis supported a time-varying association between concurrent hs-cTnI and ACR, it does not validate a specific temporal threshold for clinical decision-making. The late-period finding therefore remains hypothesis-generating and requires external validation.

## 5. Conclusions

In this single-center cohort, time-normalized hs-cTnI kinetics did not provide incremental diagnostic value beyond the concurrent hs-cTnI concentration for detecting biopsy-confirmed ACR grade ≥ 2R. Continuous-time modeling demonstrated that the association between concurrent hs-cTnI and ACR strengthened with increasing time after transplantation, whereas the kinetic term remained non-informative after accounting for this time dependence. Exploratory threshold and decision-curve analyses suggested potentially favorable late-period rule-out characteristics of concurrent hs-cTnI, but imperfect calibration and the absence of external threshold validation preclude its use as a stand-alone replacement for EMB. These findings support further prospective evaluation of concurrent hs-cTnI as an adjunctive late-period marker rather than the use of serial hs-cTnI kinetics for rejection surveillance.

## Figures and Tables

**Table 1:** Baseline characteristics of study population.

Characteristic	No ACR ≥ 2R *n* = 63	≥1 ACR ≥ 2R *n* = 76	*p*-Value
Age at OHT, years	51.8 ± 11.7	54.0 ± 12.8	0.294
Male sex	52 (82.5%)	60 (78.9%)	0.670
Included biopsy episodes per patient	9.0 (5.5–11.0)	9.5 (8.0–11.3)	0.015
Time from OHT to first included biopsy, days	14.0 (14.0–17.5)	14.0 (13.8–15.0)	0.247
Time from OHT to last included biopsy, days	363 (120–551)	363 (190–546)	0.725
Analytical observation span, days	347 (92–539)	346 (176–532)	0.631

Values are presented as mean ± standard deviation, median (interquartile range), or *n* (%), as appropriate. Patients were classified according to the occurrence of at least one ACR grade ≥ 2R episode among biopsies included in the comparative analytical dataset. Continuous variables were compared using Welch’s *t* test or the Mann–Whitney U test, as appropriate, and categorical variables using Fisher’s exact test. ACR, acute cellular rejection; OHT, orthotopic heart transplantation; SD, standard deviation.

**Table 2:** AUC values for models M1 and M2.

Period	M1	M2 (Kinetics)	ΔAUC M2–M1
All biopsies	0.641 (0.592–0.689)	0.635 (0.584–0.685)	−0.007 (−0.021–0.007)
≤90 days	0.503 (0.439–0.573)	0.494 (0.425–0.568)	−0.009 (−0.032–0.013)
>90 days	0.758 (0.664–0.838)	0.746 (0.654–0.828)	−0.012 (−0.037–0.008)

Values are presented as AUC (95% CI). M1 included concurrent log_2_-transformed hs-cTnI as the fixed-effect predictor, whereas M2 additionally included the time-normalized change in log_2_-transformed hs-cTnI per 7 days. ΔAUC was calculated as AUC for M2 minus AUC for M1. All AUC estimates were based on leave-one-patient-out cross-validated predictions, and 95% CIs were obtained using patient-level cluster bootstrap resampling. Early and late periods were defined as ≤90 and >90 days after OHT, respectively. Abbreviations: AUC, area under the receiver operating characteristic curve; CI, confidence interval; hs-cTnI, high-sensitivity cardiac troponin I; OHT, orthotopic heart transplantation.

**Table 3:** Full-data mixed-effects logistic regression models for ACR grade ≥ 2R.

Model	Effect	β	SE	OR	95% CI for OR	*p*-Value
M1	Intercept	−3.297	0.260	0.037	0.022–0.062	<0.001
M1	Concurrent log_2_(hs-cTnI)	0.194	0.038	1.214	1.127–1.309	<0.001
M2	Intercept	−3.316	0.263	0.036	0.022–0.061	<0.001
M2	Concurrent log_2_(hs-cTnI)	0.210	0.041	1.234	1.139–1.336	<0.001
M2	Time-normalized log_2_(hs-cTnI) change per 7 days	0.095	0.083	1.099	0.935–1.293	0.253

β, fixed-effect coefficient on the log-odds scale; SE, standard error; OR, odds ratio; CI, confidence interval; hs-cTnI, high-sensitivity cardiac troponin I. For concurrent log_2_(cTnI), the OR represents the change in the odds of ACR grade ≥ 2R associated with a two-fold increase in cTnI. For the kinetic term, a one-unit increase represents a time-normalized increase of one log_2_ unit over 7 days. The patient-level random-intercept variance was 0.431 in M1 and 0.446 in M2; the corresponding standard deviations were 0.657 and 0.668.

**Table 4:** Association between concurrent hs-cTnI and ACR Grade ≥ 2R at selected times after OHT in the continuous-time interaction model.

Time After OHT	OR/hs-cTnI Doubling	95% CI	*p*-Value	Summary
30 days	1.089	0.961–1.235	0.182	Not significant
90 days	1.362	1.213–1.530	<0.001	Significant
180 days	1.569	1.358–1.813	<0.001	Significant
365 days	1.812	1.502–2.187	<0.001	Significant

Abbreviations: ACR, acute cellular rejection; CI, confidence interval; hs-cTnI, high-sensitivity cardiac troponin I; OHT, orthotopic heart transplantation; OR, odds ratio.

**Table 5:** Cross-validated calibration and Brier scores for models M1 and M2 by post-transplant period.

Period	Model	Brier Score	Calibration Intercept	Calibration Slope	Interpretation
All	M1	0.0917 (0.0758–0.1082)	0.165 (−0.059–0.373)	0.869 (0.559–1.213)	Overall calibration imperfect but broadly acceptable
All	M2	0.0917 (0.0759–0.1083)	0.170 (−0.055–0.379)	0.813 (0.498–1.149)	Overall calibration imperfect but broadly acceptable
Early	M1	0.1052 (0.0859–0.1259)	0.041 (−0.234–0.289)	0.093 (−0.399–0.591)	Slope near zero; little predictive information
Early	M2	0.1058 (0.0863–0.1264)	0.065 (−0.217–0.316)	0.023 (−0.472–0.496)	Slope near zero; little predictive information
Late	M1	0.0779 (0.0554–0.1016)	0.343 (0.037–0.649)	2.454 (1.689–3.415)	Slope > 1; period-specific miscalibration
Late	M2	0.0774 (0.0551–0.1010)	0.318 (−0.065–0.625)	2.122 (1.449–2.956)	Slope > 1; period-specific miscalibration

Abbreviations: CI, confidence interval; hs-cTnI, high-sensitivity cardiac troponin I; M1, model including concurrent hs-cTnI; M2, model additionally including the time-normalized hs-cTnI kinetic term.

**Table 6:** Diagnostic performance of models M1 and M2 at an Exploratory 5% predicted-risk threshold by post-transplant period.

Period	Model	Sensitivity	Specificity	PPV	NPV	Biopsies Avoided	Missed ACR	FN/Total ACR
All	M1	97.7% (93.6–100.0)	19.7% (15.0–24.3)	12.3% (10.1–14.5)	98.6% (96.4–100.0)	17.9% (13.7–22.2)	2.3% (0.0–6.4)	3/128
All	M2	96.1% (91.9–99.2)	21.1% (16.5–25.8)	12.3% (10.1–14.6)	97.9% (95.6–99.6)	19.3% (15.0–23.6)	3.9% (0.8–8.1)	5/128
Early	M1	100.0% (100.0–100.0)	2.7% (1.1–4.8)	12.0% (9.4–14.6)	100.0% (100.0–100.0)	2.4% (1.0–4.2)	0.0% (0.0–0.0)	0/73
Early	M2	100.0% (100.0–100.0)	3.6% (1.7–6.0)	12.1% (9.5–14.7)	100.0% (100.0–100.0)	3.2% (1.5–5.3)	0.0% (0.0–0.0)	0/73
Late	M1	94.5% (85.7–100.0)	36.6% (28.5–44.8)	12.8% (9.1–16.9)	98.6% (96.2–100.0)	33.8% (26.0–41.4)	5.5% (0.0–14.3)	3/55
Late	M2	90.9% (81.2–98.3)	38.4% (30.7–46.2)	12.7% (9.0–16.9)	97.7% (95.2–99.6)	35.8% (28.4–43.4)	9.1% (1.7–18.8)	5/55

Diagnostic performance was evaluated at an exploratory predicted-risk threshold of 5%. Biopsy episodes with a predicted probability below 5% were classified as test-negative and represent the proportion of potentially avoidable biopsies. Missed ACR represents false-negative biopsy episodes with ACR grade ≥ 2R and is presented as the proportion and number of false-negative episodes among all ACR grade ≥ 2R episodes. M1 included concurrent log_2_-transformed hs-cTnI, whereas M2 additionally included the time-normalized change in log_2_-transformed hs-cTnI per 7 days. Early and late periods were defined as ≤90 and >90 days after OHT, respectively. Values in parentheses represent 95% CIs obtained using patient-level cluster bootstrap resampling. Abbreviations: ACR, acute cellular rejection; CI, confidence interval; FN, false negative; hs-cTnI, high-sensitivity cardiac troponin I; NPV, negative predictive value; OHT, orthotopic heart transplantation; PPV, positive predictive value.

## Data Availability

The data supporting the findings of this study are not publicly available due to patient confidentiality, ethical restrictions, and institutional data protection regulations. Access to appropriately anonymized data may be considered upon reasonable request to the corresponding author and subject to approval by the relevant institution and ethics committee.

## Supplementary Materials

The following supporting information can be downloaded at: https://www.mdpi.com/article/10.3390/jcm15166444/s1, Figure S1: Leave-one-patient-out cross-validated calibration plots for models M1 and M2 in the overall analytical cohort; Figure S2: Leave-one-patient-out cross-validated calibration plots for models M1 and M2 during the early post-transplant period (≤90 days after OHT); Figure S3: Leave-one-patient-out cross-validated calibration plots for models M1 and M2 during the late post-transplant period (>90 days after OHT); Table S1: Diagnostic performance across exploratory predicted-risk thresholds.
