# Supplementary material for: Serial Cardiac Troponin I Kinetics Do Not Improve Detection of Acute Cellular Rejection Beyond Concurrent Troponin Levels After Heart Transplantation
Source: J Clin Med. 2026 Aug 20;15(16):6444. doi: 10.3390/jcm15166444 (PMC13513452; doi:10.3390/jcm15166444)
Supplement: Supplementary file 1 [file jcm-15-06444-s001.zip › jcm-4477346-supplementary.pdf]

Supplementary Materials

Serial Cardiac Troponin I Kinetics Do Not Improve Detection of Acute Cellular Rejection Beyond Concurrent Troponin Levels After Heart Transplantation

M1 included concurrent log_2-transformed hs-cTnI. M2 additionally included the time-normalized change in log_2-transformed hs-cTnI per 7 days. Early and late periods were defined as ≤90 and >90 days after OHT, respectively.

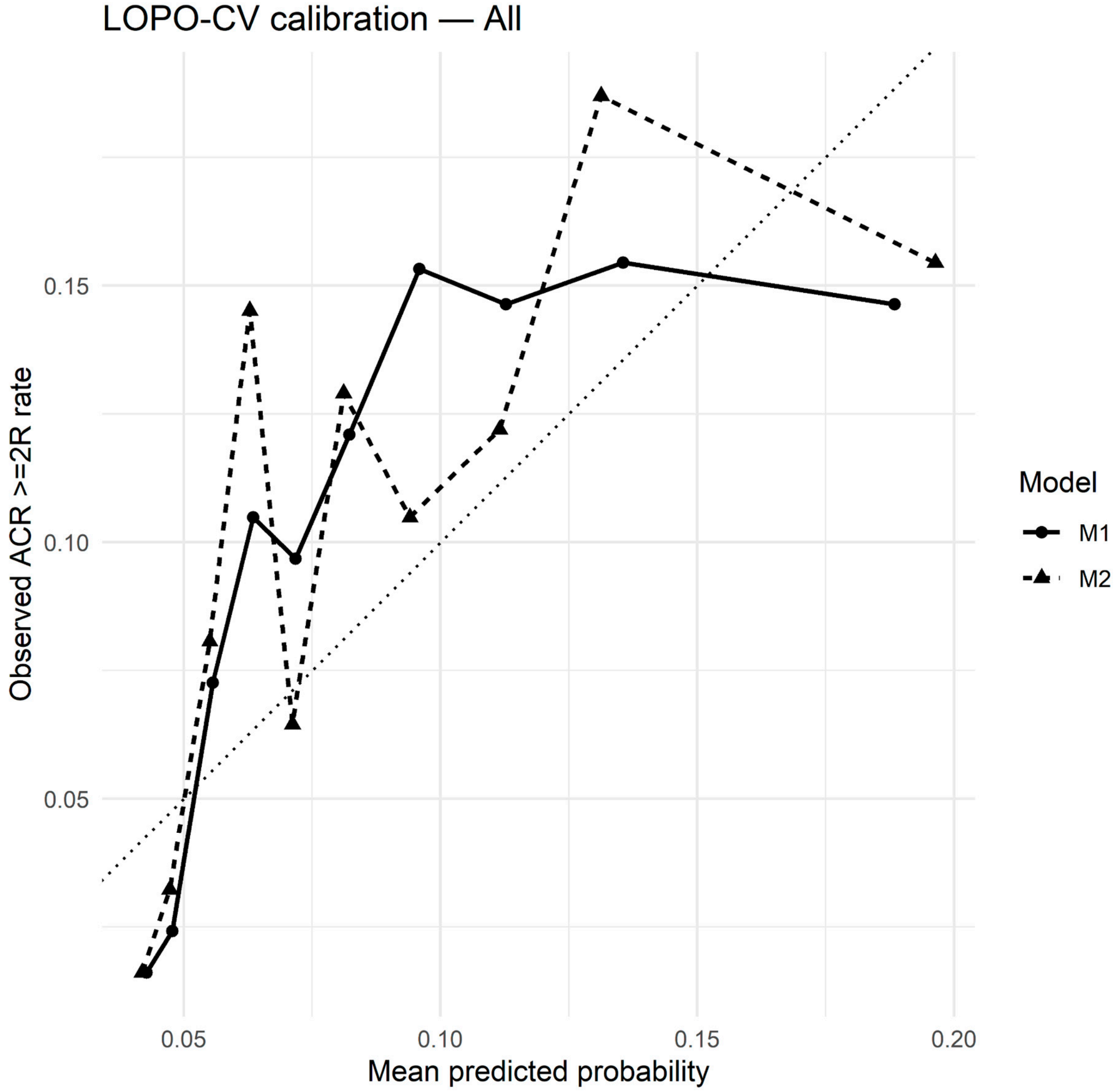

Figure S1. Leave-one-patient-out cross-validated calibration plots for models M1 and M2 in the overall analytical cohort.

The dotted diagonal represents perfect calibration.

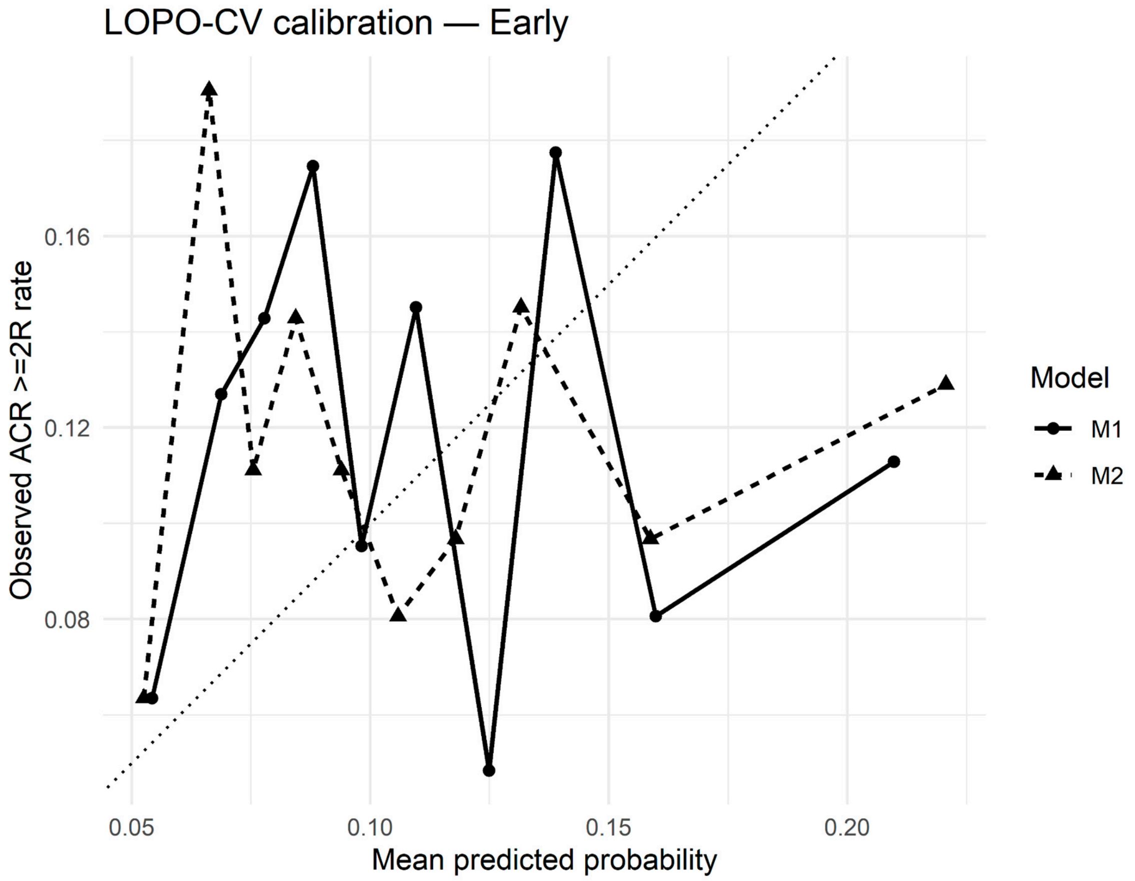

Figure S2. Leave-one-patient-out cross-validated calibration plots for models M1 and M2 during the early post-transplant period (≤90 days after OHT).

The dotted diagonal represents perfect calibration.

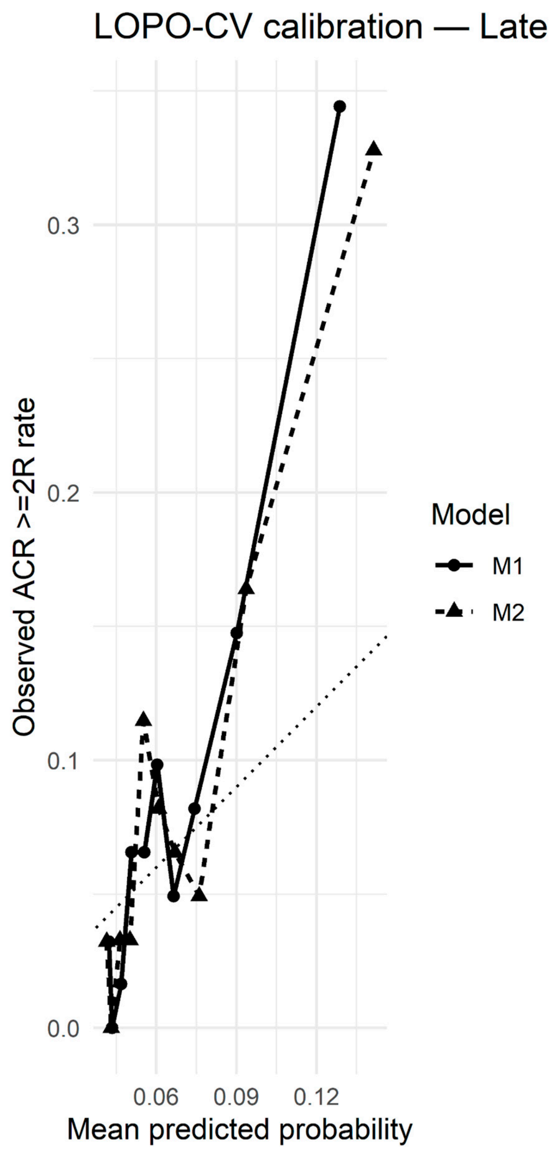

Figure S3. Leave-one-patient-out cross-validated calibration plots for models M1 and M2 during the late post-transplant period (>90 days after OHT).

The dotted diagonal represents perfect calibration.

Table S1. Diagnostic performance across exploratory predicted-risk thresholds.

Diagnostic performance was evaluated at exploratory predicted-risk thresholds of 2%, 5%, 10%, 15%, and 20%. Values are percentages with 95% confidence intervals obtained using patient-level cluster bootstrap resampling. Biopsies potentially avoided denotes biopsy episodes classified as test-negative. Missed ACR denotes false-negative episodes among all ACR grade ≥2R episodes. NPV is reported as not estimable (NA) when a threshold produced no test-negative classifications.

Period	Model	Threshold	Sensitivity (95% CI)	Specificity (95% CI)	PPV (95% CI)	NPV (95% CI)	Biopsies potentially avoided (95% CI)	Missed ACR (95% CI); FN/total ACR
All	M1	2%	100.0% (100.0–100.0)	0.0% (0.0–0.0)	10.3% (8.4–12.3)	NA	0.0% (0.0–0.0)	0.0% (0.0–0.0); 0/128
All	M1	5%	97.7% (93.6–100.0)	19.7% (15.0–24.3)	12.3% (10.1–14.5)	98.6% (96.4–100.0)	17.9% (13.7–22.2)	2.3% (0.0–6.4); 3/128
All	M1	10%	46.1% (36.1–57.6)	69.3% (65.2–73.1)	14.8% (11.3–18.4)	91.8% (89.5–94.1)	67.7% (63.4–71.6)	53.9% (42.4–63.9); 69/128
All	M1	15%	14.8% (9.0–22.0)	90.2% (87.9–92.2)	14.8% (9.5–20.6)	90.2% (88.0–92.2)	89.7% (87.3–91.8)	85.2% (78.0–91.0); 109/128
All	M1	20%	4.7% (1.5–8.9)	97.7% (96.6–98.6)	18.8% (6.9–31.6)	89.9% (87.8–91.8)	97.4% (96.3–98.4)	95.3% (91.1–98.5); 122/128
All	M2	2%	100.0% (100.0–100.0)	0.0% (0.0–0.0)	10.3% (8.4–12.3)	NA	0.0% (0.0–0.0)	0.0% (0.0–0.0); 0/128
All	M2	5%	96.1% (91.9–99.2)	21.1% (16.5–25.8)	12.3% (10.1–14.6)	97.9% (95.6–99.6)	19.3% (15.0–23.6)	3.9% (0.8–8.1); 5/128
All	M2	10%	45.3% (35.4–56.1)	70.1% (65.7–74.0)	14.9% (11.1–18.8)	91.7% (89.5–94.0)	68.5% (64.0–72.5)	54.7% (43.9–64.6); 70/128
All	M2	15%	14.8% (8.3–22.8)	91.1% (88.6–93.3)	16.1% (9.8–23.1)	90.3% (88.1–92.3)	90.5% (87.8–92.7)	85.2% (77.2–91.7); 109/128
All	M2	20%	4.7% (1.5–9.0)	96.9% (95.7–98.1)	15.0% (5.6–24.4)	89.8% (87.8–91.8)	96.8% (95.4–98.0)	95.3% (91.0–98.5); 122/128
Early	M1	2%	100.0% (100.0–100.0)	0.0% (0.0–0.0)	11.7% (9.1–14.2)	NA	0.0% (0.0–0.0)	0.0% (0.0–0.0); 0/73
Early	M1	5%	100.0% (100.0–100.0)	2.7% (1.1–4.8)	12.0% (9.4–14.6)	100.0% (100.0–100.0)	2.4% (1.0–4.2)	0.0% (0.0–0.0); 0/73
Early	M1	10%	50.7% (39.2–63.2)	46.6% (41.4–51.5)	11.1% (8.0–14.3)	87.7% (83.8–91.7)	46.9% (41.6–51.9)	49.3% (36.8–60.8); 36/73
Early	M1	15%	16.4% (8.8–25.9)	80.8% (76.6–84.7)	10.2% (5.4–15.7)	88.0% (85.0–90.9)	81.1% (77.3–84.8)	83.6% (74.1–91.2); 61/73
Early	M1	20%	6.8% (1.5–13.5)	95.7% (93.7–97.4)	17.2% (4.5–30.8)	88.6% (85.9–91.2)	95.4% (93.4–97.2)	93.2% (86.5–98.5); 68/73
Early	M2	2%	100.0% (100.0–100.0)	0.0% (0.0–0.0)	11.7% (9.1–14.2)	NA	0.0% (0.0–0.0)	0.0% (0.0–0.0); 0/73
Early	M2	5%	100.0% (100.0–100.0)	3.6% (1.7–6.0)	12.1% (9.5–14.7)	100.0% (100.0–100.0)	3.2% (1.5–5.3)	0.0% (0.0–0.0); 0/73
Early	M2	10%	46.6% (35.5–58.9)	49.5% (43.9–54.9)	10.9% (7.7–14.2)	87.5% (83.5–91.4)	49.9% (44.3–55.2)	53.4% (41.1–64.5); 39/73
Early	M2	15%	16.4% (8.3–26.2)	83.3% (78.9–87.5)	11.5% (6.1–17.6)	88.3% (85.2–91.2)	83.4% (78.9–87.4)	83.6% (73.8–91.7); 61/73
Early	M2	20%	5.5% (1.3–11.6)	94.2% (91.8–96.4)	11.1% (2.9–20.0)	88.3% (85.7–90.9)	94.2% (91.7–96.5)	94.5% (88.4–98.7); 69/73
Late	M1	2%	100.0% (100.0–100.0)	0.0% (0.0–0.0)	9.0% (6.3–12.0)	NA	0.0% (0.0–0.0)	0.0% (0.0–0.0); 0/55
Late	M1	5%	94.5% (85.7–100.0)	36.6% (28.5–44.8)	12.8% (9.1–16.9)	98.6% (96.2–100.0)	33.8% (26.0–41.4)	5.5% (0.0–14.3); 3/55
Late	M1	10%	40.0% (26.6–55.3)	91.9% (88.3–94.9)	32.8% (21.3–46.8)	93.9% (91.6–96.2)	89.1% (85.0–92.5)	60.0% (44.7–73.4); 33/55
Late	M1	15%	12.7% (4.0–23.7)	99.5% (98.7–100.0)	70.0% (44.4–100.0)	92.0% (89.1–94.6)	98.4% (96.9–99.5)	87.3% (76.3–96.0); 48/55
Late	M1	20%	1.8% (0.0–6.1)	99.6% (99.1–100.0)	33.3% (0.0–100.0)	91.1% (88.2–93.8)	99.5% (98.9–100.0)	98.2% (93.9–100.0); 54/55
Late	M2	2%	100.0% (100.0–100.0)	0.0% (0.0–0.0)	9.0% (6.3–12.0)	NA	0.0% (0.0–0.0)	0.0% (0.0–0.0); 0/55
Late	M2	5%	90.9% (81.2–98.3)	38.4% (30.7–46.2)	12.7% (9.0–16.9)	97.7% (95.2–99.6)	35.8% (28.4–43.4)	9.1% (1.7–18.8); 5/55
Late	M2	10%	43.6% (30.0–58.0)	90.5% (86.5–94.1)	31.2% (20.7–44.1)	94.2% (91.8–96.4)	87.4% (83.0–91.3)	56.4% (42.0–70.0); 31/55
Late	M2	15%	12.7% (4.0–23.7)	98.7% (97.6–99.7)	50.0% (25.0–80.0)	92.0% (89.0–94.5)	97.7% (96.0–99.1)	87.3% (76.3–96.0); 48/55
Late	M2	20%	3.6% (0.0–9.5)	99.6% (99.1–100.0)	50.0% (0.0–100.0)	91.3% (88.3–93.9)	99.3% (98.5–100.0)	96.4% (90.5–100.0); 53/55

Abbreviations: ACR, acute cellular rejection; CI, confidence interval; FN, false negative; hs-cTnI, high-sensitivity cardiac troponin I; NA, not estimable; NPV, negative predictive value; OHT, orthotopic heart transplantation; PPV, positive predictive value.
